# Mulberry bodies in the urine sediment of a patient with chronic kidney disease

**DOI:** 10.1515/almed-2020-0028

**Published:** 2020-05-05

**Authors:** Carlos Martínez-Figueroa, Karen Cortés-Sarabia, Hilda Guadalupe Catalán-Nájera, Micaela Martínez-Alarcón

**Affiliations:** Emergency Department, Clinical Laboratory, Clínica Hospital ISSSTE, Iguala, Guerrero, Mexico; Laboratory of Immunobiology and Molecular Diagnosis, Faculty of Biochemistry, UAGro, Chilpancingo, Guerrero, Mexico; Emergency Department, Clinical Laboratory, Clínica Hospital ISSSTE, Heroico Colegio Militar 3, Centro, 40000 Iguala de la Independencia, Guerrero, Mexico

**Keywords:** chronic kidney disease, fabry disease, mulberry bodies

## Abstract

**Objectives:**

Fabry disease is a hereditary disease caused by a mutation in the *α*-galactosidase A (GLA) gene resulting in the accumulation of glycosphingolipids in different organs. Timely diagnosis is crucial for the early initiation of treatment to avoid organic dysfunction secondary to lipid accumulation. In view of the above, a number of studies have been performed to assess the role of mulberry bodies as a new diagnostic tool. In this study, we report a case demonstrating the utility of this test.

**Case presentation:**

We report the case of a woman of advanced age without a history of chronic disease with symptoms consistent with urinary tract infection (dysuria, pelvic pain, and frequent urination). Based on laboratory test results, a diagnosis of anemia with concomitant chronic kidney disease was established. Urine test revealed microhematuria, proteinuria, urine sediment, and the presence of lipid particles consistent with mulberry bodies.

**Conclusions:**

The identification of mulberry bodies and cells in urine sediment is an easy-to-use tool potentially useful in diagnosing Fabry disease, which may contribute to initiate enzyme replacement therapy in a timely manner and reduce systemic deterioration.

## Introduction

Fabry disease (FD) is a systemic X-linked condition caused by a mutation in the *GLA* gene that results in a deficiency of the enzyme *α*-galactosidase (a-GAL A), thereby causing glycosphingolipids and globotriaosylceramide (GL-3) to progressively buildup in the endothelium of the heart, central nervous system, and kidneys [1], [2]. This disease manifests mostly in hemizygous males in the form of cardiovascular disease, end-stage renal disease, and cerebrovascular events. In contrast, heterozygous females remain asymptomatic or exhibit milder symptoms [3]. FD diagnosis is based on a-GAL A determination in plasma. However, there are recent reports of mulberry bodies and cells in urine sediment [2]. We describe the case of a patient with FD who presented these structures.

## Case presentation

**Figure 1:**
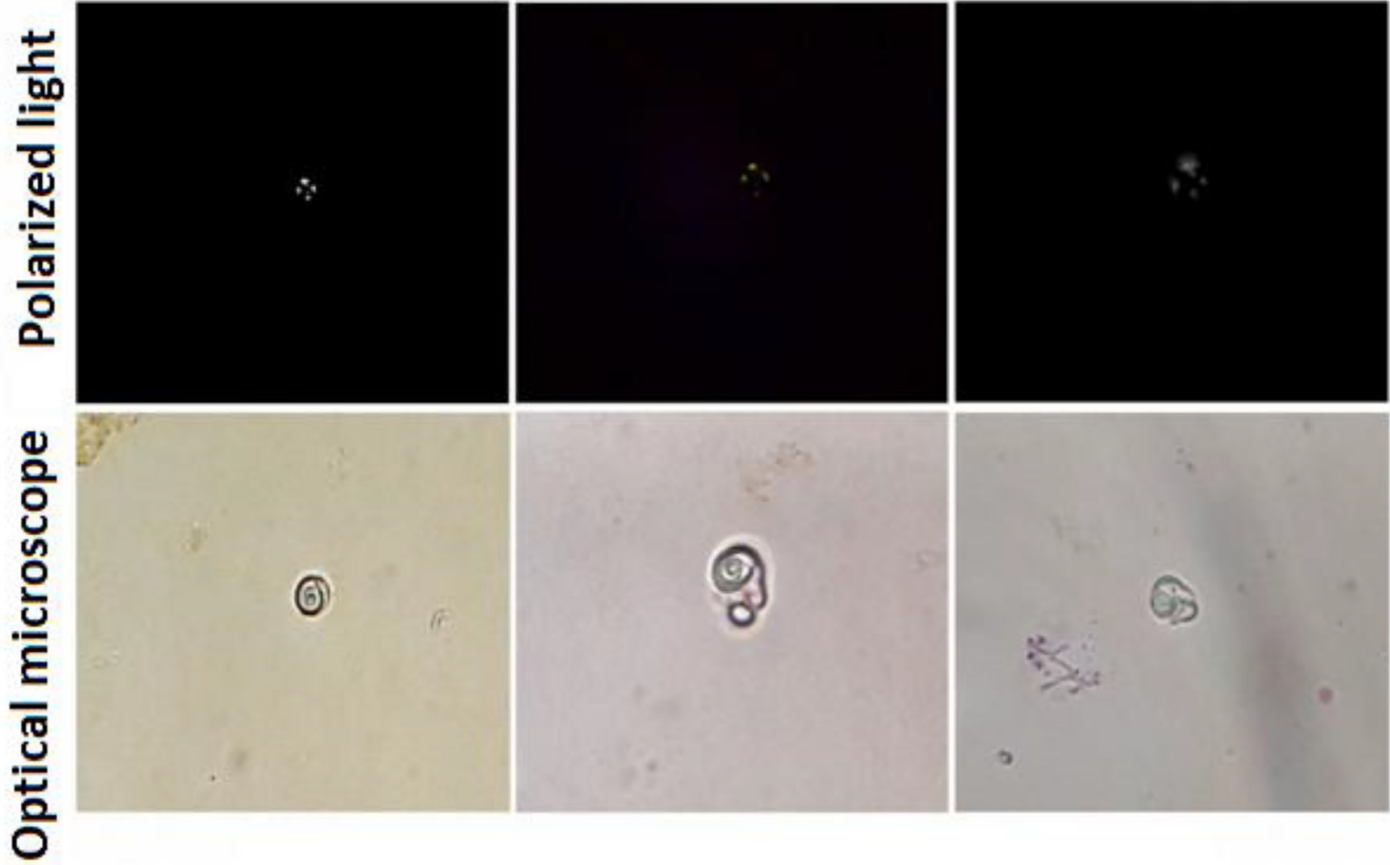
Mulberry bodies in urine sediment. Upper side: Maltese-cross patterned mulberry bodies on polarized light 40×. Lower side: Atypical lipid structures on bright-field microscopy 40×.

We report the case of an 88 year-old patient who presented to the emergency department with pelvic pain, dysuria, and frequent urination. A diagnosis of urinary tract infection was established. In the lack of a medical record, laboratories tests were requested. Blood cytometry (Swelab Alfa Basic, Boule Diagnostics) showed a hemoglobin level of 8.9 g/dL (12–15 g/dL); hematocrit 28% (38–47%); leukocytes 14.14 × 10^3^/mm^3^ (5–10 × 10^3^/mm^3^) and platelets 573 × 10^3^/mm^3^ (150–400 10^3^/mm^3^). Blood chemistry (RX Daytona, RANDOX) showed a glucose level of 76 mg/dL (70–110 mg/dL); urea 97 mg/dL (10–50 mg/dL); creatinine 1.6 mg/dL (0.6–1.3 mg/dL); sodium 153 mmol/L (135–148 mmol/L); potassium 4.2 mmol/L (3.5–5.3 mmol/L); and chlorine 95 mmol/L (98–107 mmol/L). Glomerular filtration rate (GFR) yielded values of 30.5 mL/min/1.73 m^2^. Urine test revealed a density of 1,010, pH 8.5, trace proteins, hemoglobin (25 red blood cells/µL) and leukocyte esterase (15 white blood cells/µL). Urinary sediment examination showed hematuria (4–6 red blood cells / field), few granular cylinders, and scarce bacteria and squamous and cylindrical cells. The presence of spiral-shaped lipid structures was confirmed by the identification of a Maltese cross pattern on polarized light examination. These structures were consistent with the mulberry bodies that are pathognomonic of FB (Figure 1), which led to identify Fabry nephropathy as the origin of chronic kidney disease.

## Discussion

Patients with FD typically develop a nephropathy, with albuminuria during childhood being an early sign, and proteinuria occurring as patients approach their 20 and 30s. By the age of 50, many have developed end-stage renal disease [4]. Early diagnosis is crucial to starting enzyme replacement therapy and preventing the progression of FD-related conditions. However, some FD patients may exhibit atypical symptoms or show alterations in a specific organ resulting in cardiac and renal disease [2], [4]. The case reported here did not show typical symptoms, but exhibited an impaired renal function evidenced by elevated levels of urea and creatinine, and depressed levels of TFG. These values are classified as G3B stage (chronic kidney disease) according to KDIGO guidelines [5]. The patient had no history of type 1 or 2 diabetes mellitus, which is one of the main causes of kidney disease. Blood cytometry revealed anemia, which may be secondary to chronic kidney disease. The structures observed in urine sediment, identified as mulberry bodies, originated from mulberry cells, which are convoluted tubule cells with accumulated globotriaosylceramide containing lamellar, spiral-shaped micelles. These particles are a frequent finding in FD patients [6]. Selvarajah et al. investigated mulberry bodies as a marker of FD. The authors reported that the presence of these structures has a sensitivity and a specificity of 100% in the diagnosis of FD, as it is present in patients with and without nephropathy [7]. In view of the aforementioned mulberry cells have been proposed as a marker of FD, when enzyme assays cannot be performed and upon suspicion of the disease. In conclusion, the identification of mulberry bodies in urine sediment is confirmatory of FD and warrants the timely initiation of treatment [6], [8], [9].

### Five lessons learned

– FD results from a deficiency in the *α*-galactosidase A.
– The accumulation of glycosphingolipids in the kidneys causes chronic kidney disease.
– Mulberry bodies have a specificity and sensitivity of 100% in the diagnosis of FD.
– Mulberry bodies are lipid structures containing lamellar, spiral-shaped bodies.
– Mulberry bodies and cells form before the FD patient develops kidney disease.
